# ‘Cool and quiet’ therapy for malignant hyperthermia following severe traumatic brain injury: A preliminary clinical approach

**DOI:** 10.3892/etm.2014.2130

**Published:** 2014-12-15

**Authors:** YU-HE LIU, ZHEN-DE SHANG, CHAO CHEN, NAN LU, QI-FENG LIU, MING LIU, JING YAN

**Affiliations:** Department of Neurosurgery, The 88th Hospital of PLA, Taian, Shandong 271000, P.R. China

**Keywords:** traumatic brain injury, malignant hyperthermia, mild hypothermia, ‘cool and quiet’ therapy

## Abstract

Malignant hyperthermia increases mortality and disability in patients with brain trauma. A clinical treatment for malignant hyperthermia following severe traumatic brain injury, termed ‘cool and quiet’ therapy by the authors of the current study, was investigated. Between June 2003 and June 2013, 110 consecutive patients with malignant hyperthermia following severe traumatic brain injury were treated using mild hypothermia (35–36°C) associated with small doses of sedative and muscle relaxant. Physiological parameters and intracranial pressure were monitored, and the patients slowly rewarmed following the maintenance of mild hypothermia for 3–12 days. Consecutive patients who had undergone normothermia therapy were retrospectively analyzed as the control. In the mild hypothermia group, the recovery rate was 54.5%, the mortality rate was 22.7%, and the severe and mild disability rates were 11.8 and 10.9%, respectively. The mortality rate of the patients, particularly that of patients with a Glasgow Coma Scale (GCS) score of between 3 and 5 differed significantly between the hypothermia group and the normothermia group (P<0.05). The mortality of patients with a GCS score of between 6 and 8 was not significantly different between the two groups (P> 0.05). The therapy using mild hypothermia with a combination of sedative and muscle relaxant was beneficial in decreasing the mortality of patients with malignant hyperthermia following severe traumatic brain injury, particularly in patients with a GCS score within the range 3–5 on admission. The therapy was found to be safe, effective and convenient. However, rigorous clinical trials are required to provide evidence of the effectiveness of ‘cool and quiet’ therapy for hyperthermia.

## Introduction

Malignant hyperthermia following severe traumatic brain injury occurs due to damage to the thermoregulatory centers, occurring within the first three days after head trauma, a time frame less likely for hyperthermia to be attributable to infectious causes (1). Previous studies have shown that malignant hyperthermia increases mortality and disability in patients with brain trauma (1–5). In brain damage such as stroke, hyperthermia acts through several mechanisms to exacerbate cerebral ischemia (1), including the increased release of neurotransmitters, excessive production of oxygen radicals, extensive blood-brain barrier breakdown, increased ischemic depolarizations in the focal ischemic penumbra, impaired recovery of energy metabolism, enhanced inhibition of protein kinases and worsening of cytoskeletal proteolysis (6,7). Hyperthermia significantly increases the incidence of infection (1) and elevates the intracranial pressure, causing brain cell damage (4). Hyperthermia can increase the metabolism of the body, accelerate organ failure and affect the efficacy of neuroprotectant and thrombolytic therapy (8,9). Therefore, the control of hyperthermia is necessary in the treatment of traumatic brain injury. Therapeutic hypothermia has become a focus of research in recent years.

Previous studies have shown that hypothermia can reduce the basal metabolic rate, the consumption of oxygen by brain cells (5,10) and intracranial pressure, and protect the blood-brain barrier. Hypothermia has neuroprotective effects (11), which involve reduced extracellular glutamate release (12–14), limited calcium transfer (15), the reduction of free radicals (12), the inhibition of nitric oxide (16,17) and reduced brain metabolism. However, the lower the temperature, the greater the incidence of side-effects and complications (18), such as shivering, reduced electrolyte levels, dysregulated acid-base status, insulin resistance, kidney dysfunction, arrhythmia and impaired immune function. Currently, the temperature range of therapeutic hypothermia remains controversial (14). A number of studies have described the effects of moderate hypothermia (32–35°C); however, due to the various complications (19), difficulties in temperature maintenance and damage following rewarming (20), the clinical application of hypothermia is limited. Certain studies have demonstrated that mild hypothermia can help to improve outcomes (21,22) without clear explanation. Thus, it is essential to balance the maximum efficacy and minimum complications of therapeutic hypothermia. The aim of the present study was to investigate a new therapeutic hypothermia method known as ‘cool and quiet’ therapy for malignant hyperthermia in patients following severe traumatic brain injury

## Patients and methods

### Patient selection

A total of 110 consecutive patients in the 88th Hospital of PLA (Taian, China) with malignant hyperthermia following severe traumatic brain injury were enrolled from June 2003 to June 2013. The patients had a Glasgow Coma Scale (GCS) score of between 3 and 8 points, had spent >6 h in a coma after injury, or experienced a deterioration of awareness following >6 h in a coma within 24 h after injury. Cases with serious infections, dehydration fever, transfusion reactions, use of psychotropic inhibitors or clinical brain death (GCS ≤3 and no brain-stem reflexes) were excluded. In addition, 110 cases that had undergone normothermia therapy, in which patients’ temperatures were maintained between 36–37°C, were retrospectively analyzed as the control. The present study was conducted according to the revised Declaration of Helsinki (2008 edition), and the approval of the ethical committee of the 88th Hospital of PLA was obtained. Written informed consent was provided by all participants prior to the study.

### Patient assessment

Patients who met the entry criteria were examined by a continuous CT scan, and the GCS was determined based on ability of the patient to open their eyes, speak, and use their arms or legs. Temperature measurement methods were as follows: As there are numerous blood vessels around the rectum, which more sensitively reflect changes in body temperature, rectal temperature measurements were preferred. When using the retention enema, the temperatures of the patients were measured orally.

### Treatment protocol

On the basis of actively treating the primary disease, all patients were treated with mild hypothermia (35–36°C) in addition to small doses of sedative and muscle relaxant.

### Basic treatment

**Figure 1:**
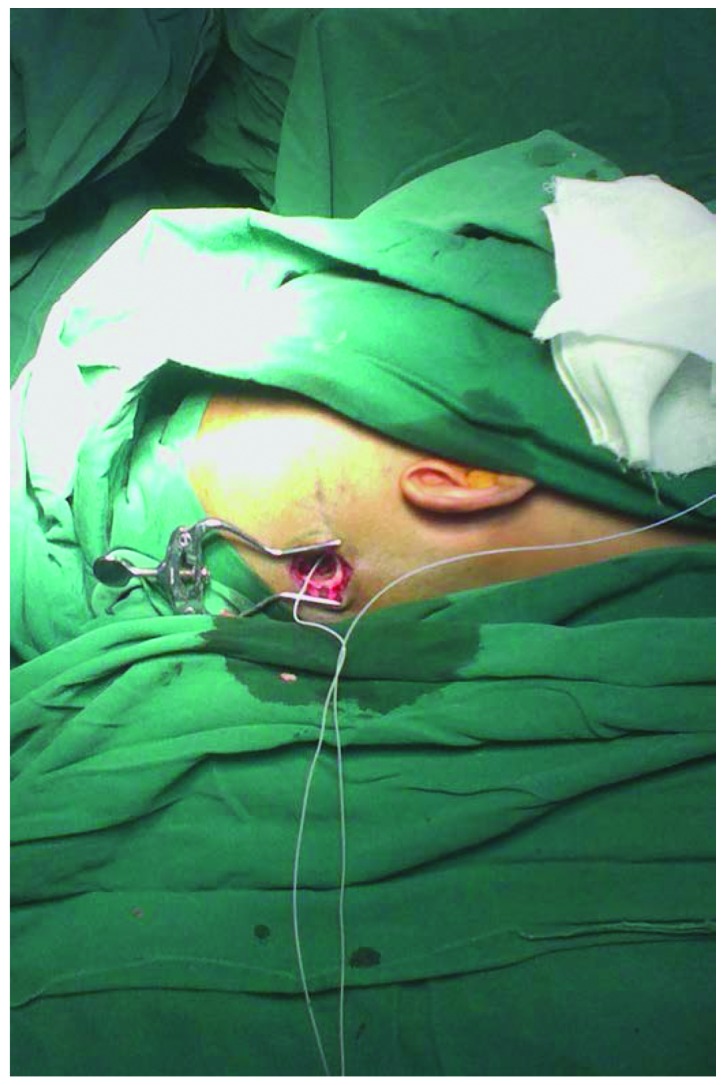
Intracranial pressure monitoring during surgery.

**Figure 2:**
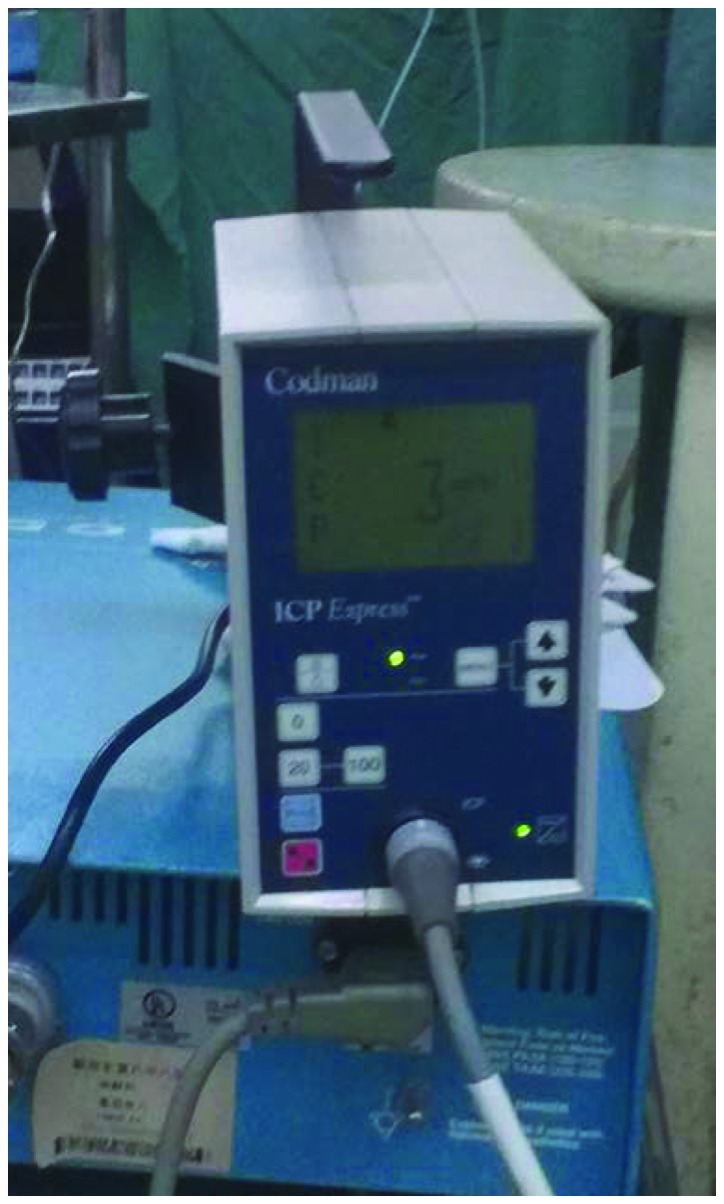
Codman intracranial pressure monitor.

On the basis of actively treating the primary trauma, patients with severe cerebral edema, extensive cerebral contusion and brainstem damage that were unconscious were treated with the programmed cooling therapy as soon as possible. It was maintained for 3–12 days, over the period at which trauma acute reactions and edema peak. The therapy was administered in combination with monitoring of the heart rate, blood pressure, breathing and pulse. The invasive intracranial pressure (ICP) of 40 patients was monitored during surgery or through a probe placed through a hole drilled in the skull (Fig. 1), with a Codman intracranial pressure monitor (Codman Neuro, Raynham, MA, USA; Fig. 2) and intracranial pressure sensor.

### Mild hypothermia with physical methods

- Hypothermia instrument such as an ice blanket. The efficacy of such a method was significant, and so was considered first. The body temperature was set to 35–36°C and the water temperature was set to 8–15°C. The room temperature was maintained at 20–25°C. When the body temperature was higher than the maximum of the set temperature, the water cycle started, which then took heat away and lowered the body temperature.
- Simple body surface hypothermia. The patient’s head (with the exception of the face) was put in an ice cap or ice tank, setting the temperature of the electronic ice cap at −2 to +2°C. Concurrently, ice salt or chemical ice bags wrapped in a towel were placed around both sides of the neck, armpits, groin and other parts of the body with large vessels. Patients, with the exception of those with brain herniation were given a retention enema with 500 ml cold saline and 1 g aspirin for 30 min. The temperature was measured again 30 min after the retention enema and the retention enema was repeated every 4–6 h. Through the above method, the temperature was maintained at 35–36°C.

### Sedative and muscle relaxant

To avoid shivering with skin reactions and to eliminate the acute stress reaction of the body to the internal and external environment, sedative and muscle relaxant administration was carried out simultaneously with hypothermia. Patients with increased intracranial pressure accompanied by restlessness, fever, convulsions and decerebrate rigidity were treated with sedative and muscle relaxant as early as possible.

Formula I (chlorpromazine, meperidine and promethazine) was applicable to patients with a high fever and dysphoria. This formula is recommended to be used with caution in patients with respiratory failure and those younger than 1 year or older than 60 years old. Formula II (chlorpromazine, promethazine and hydergine) was used to treat patients in whom the hyperthermia was accompanied by respiratory dysfunction or tachycardia. Formula III (chlorpromazine, promethazine and procaine) was administered to patients with oliguria. This formula is recommended to be used with caution in patients with a slow heart rate or arrhythmia. Formula IV (chlorpromazine and promethazine) was administered to patients with relatively mild hyperthermia.

When selecting Formula I or II, it was necessary to consider the condition of the patient. A full or half dose was required to fully achieve the requirements at the first administration, with physical cooling applied half an hour after the administration of the sedative and muscle relaxant. The dose was sufficient if patients did not undergo shivering with skin reactions; if it was insufficient, additional medication was administered. After this treatment, the temperature gradually decreased. When the desired temperature was reached, the treatment was transferred to the maintenance phase. Ice application was reduced and alternative medication at a half to a third of the full dose was administered every 4–8 h. Formula II was generally used as an additional medication, since Formula I contained meperidine which could cause inhibition of respiration.

The duration of hypothermia was 3–12 days, and rewarming was carried out every 2–3 days. The temperature was observed and therapeutic hypothermia was administered again if necessary; in addition, if inhibition of respiration occurred due to meperidine administration, the treatment was terminated. The duration of the hypothermia was extended until the edema had gone for patients with severe cerebral edema.

The rate of temperature lowering in adults was at 1–2°C/h, to the final temperature of 35–36°C, while that of children was maintained at 2–3°C/h. Rapid cooling may cause shivering and increase oxygen consumption, which should be avoided. In certain cases, corticosteroids were used at an early stage to protect the blood-brain barrier. Short-range high-dose shock therapy was used, firstly with dexamethasone and methylprednisolone. The dose of methylprednisolone was 5 mg/kg, repeated every 6 h. The dose was decreased to 1 mg/kg after 24 h and maintained for three days. Intrathecal medications were given for hemorrhagic cerebrospinal fluid replacement once a day. Hemorrhagic cerebrospinal fluid was slowly replaced at a volume of 30–50 ml each time. The cerebrospinal fluid turned clear in approximately a week.

### Statistical analysis

The Statistical Package for Social Sciences (SPSS, Inc., Chicago, IL, USA), version 16.0 was used for data analysis. Continuous variables are expressed as the mean ± standard deviation, and categorical variables are expressed as proportions. Comparisons between continuous variables were performed using independent-samples t-test or variance analysis; comparisons between categorical variables were performed by the χ^2^ test. P≤0.05 was considered to indicate a statistically significant result.

## Results

### Characteristics of the patients

A total of 110 patients were enrolled in the study, and the duration of hyperthermia ranged from 3 to 7 days. There were 42 cases with hyperthermia within 12 h after injury and 68 cases within 24 h.

There were 73 cases with extensive cerebral contusion including serious brain tissue swelling with obliteration of the basal cisterns of the brain and fissures, 44 cases with multiple intracranial hematoma, 71 cases with brainstem contusion and hemorrhage, 26 cases with diffuse axonal injuries, 38 cases with herniation, 85 cases with subarachnoid hemorrhage and 90 cases with contrecoup injuries. In addition, there were 70 cases requiring emergency craniotomy and hematoma surgery, including 34 cases with unilateral craniotomy, 35 cases with bilateral craniotomy, and one case with removal of a hematoma in three parts in three separate surgeries.

### Outcomes

Of the 110 cases treated with mild hypothermia, 25 cases succumbed following a persistent fever of >40°C, and certain cases were accompanied by severe complications, including 12 cases with acute renal function failure, 5 cases with convulsions, 15 cases with gastrointestinal bleeding, 24 cases with severe pulmonary infection, 13 cases with high blood sugar and 4 cases with pseudomembranous enteritis. A total of 85 cases survived, including 13 cases of vegetative state, 12 cases with living disability and 60 cases that were restored to a good state of health. In the mild hypothermia group, the recovery rate was 54.5% and mortality rate was 22.7%. The severe and mild disability rates were 11.8 and 10.9%, respectively.

The GCS scores and ICP data of the hypothermia group showed no significant difference compared with those of the normothermia group, (P>0.05; Tables I and II). The mortality rates of the patients in the hypothermia group and normothermia group exhibited a significant difference (P=0.038; Table III). The mortality rates of patients with GCS scores of between 3 and 5 revealed a statistically significant difference between the two groups (P=0.011; Table III). No significant difference was observed in the mortality rates of patients with GCS scores of between 6 and 8 between the two groups (P>0.05; Table III).

## Discussion

During the clinical treatment, it was observed that simply using head ice caps and ice bags on the surface of large vessels, did not provide a satisfactory cooling effect. There were four reasons: Unreasonably placed ice bags or caps, a limited contact area and a gap between the cooling device and the skin resulted in insufficient heat conduction; patients in a lateral position had a reduced contact area with the ice blanket; the cold stimulation exacerbated blood capillary contraction, which decreased heat dissipation; and shivering caused by the cold increased the production of heat. Therefore, it was considered necessary to combine this treatment with other physical cooling treatments.

A sponge bath using tepid water mixed with alcohol was found to be a simple, safe and efficacious method. Not only could the alcohol and water stimulate the capillaries in the skin to dilate and increase the heat dissipation, but also evaporation was able to take heat away. This could be used as a rapid therapeutic method during the initial stage of stepwise cooling.

The sedative and muscle relaxant were administered to reduce cellular metabolism, block the acute stress response, control muscle spasms and shivering, prevent convulsions and expand peripheral vessels. The formulations used were unrestricted; however, it is recommended that the dose and rate of administration are strictly controlled. In the present study, three cases experienced a sudden drop in blood pressure due to the intravenous therapy being administered too rapidly. Due to the reduced heart rate and blood pressure, low body temperature and weak breathing, the medication was discontinued. No fatal complications such as cardiac arrhythmia were observed during the cooling process and the related nursing care was easy to carry out. With a lower mortality rate compared with that of normothermia therapy, the mild hypothermia therapy was demonstrated to be safe, convenient and efficacious.

Short-range high-dose shock therapy with corticosteroids (23,24) acts by stabilizing ion channels in the cell membrane and promoting the outward flow of Ca^2+^ ions, thereby reducing phosphate kinase activity and cell metabolism, which is conducive to the functional recovery of the temperature regulating center. However, it is necessary to pay attention to side-effects such as gastrointestinal bleeding (25,26), glucose (27) and nitrogen metabolism disorders (28) and immunosuppression (29).

The initiation and duration times of hypothermia remain controversial (5), due to the different types and severities of traumatic brain injury. A review of 13 clinical studies (30) found that treatment was started 6–22 h from onset and was maintained for an average of 40.9 h (range, 24–67 h). The mean duration in the present study was >7 days and the longest case had a duration of 12 days; this patient awoke from a coma 36 days after the injury and the brain function recovered well.

Formula II of sedative and muscle relaxants was found to be the optimum choice. Hydergine is able to expand blood vessels, has a relatively low inhibitory effect on the respiratory center and does not inhibit the adenosine triphosphate enzyme system, which is beneficial to further regulate metabolism disorders, ensure the effective circulating blood volume of the heart, brain and kidneys and facilitate the recovery of central nervous system functioning (31).

In conclusion, therapy using mild hypothermia associated with sedative and muscle relaxant was beneficial in reducing the mortality of patients with malignant hyperthermia following severe traumatic brain injury, particularly in patients with GSC scores of between 3 and 5 on admission. Due to the low incidence of complications and ease of nursing care, the mild hypothermia therapy was considered to be efficacious, safe and convenient. However, a retrospective study was used to explore the mild hypothermia therapy, without a randomized controlled trial. Prospective rigorous randomized clinical trials are required to provide evidence of the efficacy of ‘cool and quiet’ therapy in the treatment of hyperthermia.

## Figures and Tables

**Table I:** Clinical observations of the two types of therapy.

		Prior to treatment		Following treatment	
			
Groups	Cases	T (°C)	GCS score	T (°C)	GCS score
Normothermia	110	39.54±1.9	5.7±2.1	37.37±1.2	8.4±1.8
Mild hypothermia	110	39.62±1.6	5.5±1.9	37.73±1.6	8.7±1.5

T, temperature; GCS, Glasgow Coma Scale.

**Table II:** ICP statistics of the two types of therapy.

Groups	Cases	ICP prior to treatment (mmHg)	ICP following treatment (mmHg)
Normothermia	40	26.8±17.5	12.3±8.5
Mild hypothermia	40	27.5±16.9	13.8±7.8

ICP, intracranial pressure.

**Table III:** Mortality of patients with different coma scores in the two groups.

Initial GCS group	Mild hypothermia	Normothermia	χ^2^	P-value
All patients				
Mortality, n (%)	25 (22.7)^a^	39 (35.5)	4.319	0.038
Total, n	110	110		
Patients with coma scores of 3–5				
Mortality, n (%)	18 (40.9)^a^	28 (68.3)	6.409	0.011
Total, n	44	41		
Patients with coma scores of 6–8				
Mortality, n (%)	7 (10.6)	11 (15.9)	0.831	0.362
Total, n	66	69		

a P<0.05 compared with normothermia.

GCS, Glasgow Coma Scale.
